# Untangling the Web of Systemic Autoinflammatory Diseases

**DOI:** 10.1155/2014/948154

**Published:** 2014-07-15

**Authors:** Donato Rigante, Giuseppe Lopalco, Antonio Vitale, Orso Maria Lucherini, Francesco Caso, Caterina De Clemente, Francesco Molinaro, Mario Messina, Luisa Costa, Mariangela Atteno, Franco Laghi-Pasini, Giovanni Lapadula, Mauro Galeazzi, Florenzo Iannone, Luca Cantarini

**Affiliations:** ^1^Institute of Pediatrics, Policlinico A. Gemelli, Università Cattolica Sacro Cuore, Rome, Italy; ^2^Interdisciplinary Department of Medicine, Rheumatology Unit, University of Bari, Bari, Italy; ^3^Research Center of Systemic Autoimmune and Autoinflammatory Diseases, Rheumatology Unit, Policlinico Le Scotte, University of Siena, Viale Bracci 1, 53100 Siena, Italy; ^4^Division of Pediatric Surgery, Department of Medical Sciences, Surgery, and Neuroscience, University of Siena, Siena, Italy; ^5^Rheumatology Unit, Department of Clinical Medicine and Surgery, University Federico II, Naples, Italy; ^6^Department of Medical Sciences, Surgery and Neurosciences, University of Siena, Siena, Italy

## Abstract

The innate immune system is involved in the pathophysiology of systemic autoinflammatory diseases (SAIDs), an enlarging group of disorders caused by dysregulated production of proinflammatory cytokines, such as interleukin-1*β* and tumor necrosis factor-*α*, in which autoreactive T-lymphocytes and autoantibodies are indeed absent. A widely deranged innate immunity leads to overactivity of proinflammatory cytokines and subsequent multisite inflammatory symptoms depicting various conditions, such as hereditary periodic fevers, granulomatous disorders, and pyogenic diseases, collectively described in this review. Further research should enhance our understanding of the genetics behind SAIDs, unearth triggers of inflammatory attacks, and result in improvement for their diagnosis and treatment.

## 1. Introduction

The innate immune system acts as an immunosurveillance keeper that discriminates among healthy host tissue, cellular debris, apoptotic cells, chromatin-modifiers, and transcription factors, mediating detection of infectious agents and instructing the adaptive immune response; the discovery and recent characterization of danger-sensing molecules of the innate immunity have revealed that immunologic homeostasis relies on a complex and highly integrated system of cells, complement, and defense factors with complementary specificity and action [1]. Systemic autoinflammatory diseases (SAIDs) are a growing cluster of heterogeneous disorders, characterized by apparently inexplicable recurrence of multiorgan inflammation in the absence of autoreactive T-lymphocytes and autoantibodies, involving the innate immune system; all these diseases are caused by a lack of regulation in the inflammasome, a large intracellular multiprotein platform, that plays a crucial role in innate immunity, leading to overproduction of proinflammatory cytokines, such as interleukin (IL)-1*β* and tumor necrosis factor (TNF)-*α*, and to a pathological delay in the turning off of inflammatory responses [2]. A brief summary of SAIDs described in this review can be found in Table 1.

## 2. Hereditary Periodic Fever Syndromes

The main subgroup of SAIDs includes different hereditary periodic fever syndromes (HPFs), sharing a common clinical background characterized by recurrent fever attacks and inflammation involving different sites, such as skin, serosal membranes, joints, gastrointestinal tube, or central nervous system. AA amyloidosis is their most serious long-term complication, with an overall prevalence ranging from 2 to 25% of cases [3]. HPFs include familial Mediterranean fever, associated with homozygosity or compound heterozygosity in the* MEFV* gene located on chromosome 16p13.3, encoding a 781-amino acid length protein, named pyrin (also known as marenostrin), which is mainly expressed in polymorphonuclear cells and monocytes; mevalonate kinase deficiency syndrome, also known as hyperimmunoglobulinemia-D syndrome, caused by homozygosity or compound heterozygosity for disease-causing mutations in the* MVK* gene, which has been localized to chromosome 12q24 and encodes for mevalonate kinase, a cytosolic enzyme involved in the cholesterol and isoprenoid biosynthesis pathway; TNF receptor-associated periodic syndrome, related to mutations in the soluble TNF receptor superfamily member 1A gene on chromosome 12p13; and finally the family of cryopyrin-associated periodic syndrome, which in turn includes familial cold urticaria syndrome (FCAS), Muckle-Wells syndrome (MWS) and chronic infantile neurological cutaneous and articular syndrome (CINCAs), all caused by mutations in the* NLRP3* gene, located on chromosome 1, which encodes for the cryopyrin protein [2, 4]. Familial Mediterranean fever and mevalonate kinase deficiency syndrome are transmitted by autosomal recessive inheritance, while inheritance is autosomal dominant for TNF receptor-associated periodic syndrome and cryopyrin-associated periodic syndrome. Generally speaking, genes associated with HPFs encode proteins involved in the regulation, activation, and/or deactivation of the inflammatory response, and disease flares usually alternate with symptom-free intervals of variable duration, characterized by entire wellbeing and complete normalization of acute phase reactants [4]. Clues useful for differential diagnosis of HPFs are listed in the Table 2.

*Familial Mediterranean fever* (FMF) is paradigmatic among HPFs and is characterized by recurrent acute fever episodes lasting from 1 to 3 days, in its most classic phenotype, arthritis, erysipelas-like erythema, and polyserositis presenting with abdominal and/or thoracic pain [5]. An inflammation of the testicular tunica vaginalis may also occur [6]. Muscular and skeletal involvement is frequent, often as recurrent simple myalgia or arthralgia, while approximately 30% of FMF patients complain of arthritides, especially affecting large joints, which are rarely erosive but may even persist for several days after fever resolution [7, 8]. More than 60% of patients have a disease onset before age 10 and 98% before age 30 [9, 10]. FMF is common in populations of Mediterranean ancestry and has traditionally been considered an autosomal-recessive disease, caused by mutations in the* MEFV* gene. Among almost 300 mutations discovered in the* MEFV* gene so far, five mutations (M694V, V726A, M680I, M694I, and E148Q) are the most frequent in the classically affected populations (Armenians, Arabs, Jews, and Turks). Adult-onset FMF seems to be genetically related to heterozygosity and low-penetrance mutations, although these patients often experience a milder disease course, with clinical features similar to those found in younger patients, except for a lower rate of arthritis and erysipelas-like rash [11]. Diagnosis of FMF relies on the Tel Hashomer clinical criteria, based on recurrent episodes of fever and serosal inflammation (manifested by nonspecific signs, such as sterile peritonitis and/or pleurisy) and clinical efficacy of daily oral administration of colchicine [12]. However, oligosymptomatic adult-onset patients may not fulfill diagnostic criteria, and in these cases genetic testing is useful in making a definite diagnosis [13, 14]. Genetic diagnosis of FMF can be certified if two mutations are identified on both of a patient's chromosomes, but many patients may have only one mutation or do not display any mutation at all. Given the high carrier frequency of* MEFV* mutations and the lower-than-expected prevalence of the disease, it seems possible that other alleles could modify inflammatory signals initiated by mutant pyrin. Thus, FMF may not be a simple monogenic inflammatory disease, and the FMF phenotype may occur in patients with only one* MEFV* mutation in the presence of other permissive alleles or environmental factors; genome-wide association studies might help confirming the polygenic influences on the clinical expression of FMF [15, 16]. Occasional reports of autosomal dominant FMF have also been associated with the compound pyrin variant E148Q/M694I encoded on a single allele (revealed in independent Indian and Turkish kindreds) and heterozygosity for the simple deletion mutation encoding pyrin ΔM694 (in three unrelated British families) [17]. Standard therapy for the prevention of acute FMF attacks and also FMF-related amyloidosis is colchicine [18]; febrile attacks are generally prevented by colchicine administration in most patients [19, 20]. Valid therapeutic alternatives are anti-IL-1 agents in unresponder or noncompliant patients [21, 22].

*Mevalonate kinase deficiency syndrome* (MKD) usually starts in the first year of life and is characterized by lifelong recurrent fever episodes, typically ranging from 3 to 7 days [23]. The most frequent symptoms reported during febrile attacks, sometimes precipitated by vaccination, infections, emotional stress, trauma, or surgery, are abdominal pain, diarrhea, vomiting, arthralgia, lymphadenopathy, heterogeneous skin lesions, and aphthous ulcers [24]. The disease is caused by different* MVK *mutations, which elicit clinical signs ranging from the milder hyperimmunoglobulinemia-D syndrome (HIDS) to its most severe expression, named “mevalonic aciduria.” However, autoinflammatory attacks in HIDS can even be complicated by macrophage activation syndrome [25]. Another recently-reported potential complication of MKD is retinitis pigmentosa, characterized by night blindness and peripheral vision loss, which can occur as a result of specific* MVK* mutations with mild systemic symptoms [26]. Using genome-wide exon sequencing technologies Zhang et al. have also found* MVK* mutations in patients with disseminated superficial actinic porokeratosis, a severe rare chronic cutaneous disorder with high genetic heterogeneity, which causes hyperkeratosis and noncontagious dry itchy lesions worsened by sun exposure; mevalonate might have a role in regulating calcium-induced keratinocyte differentiation and protecting keratinocytes from apoptosis induced by ultraviolet radiations [27]. As regards diagnosis of MKD, patients might display increased serum IgD concentrations in every period of life, both febrile and interfebrile, while urinary concentration of mevalonic acid is increased only during febrile flares. Genetic testing remains essential for a definite confirmation [28, 29]. To date, no single therapy has been found to be effective in the totality of MKD patients [21]. Most of them use nonsteroidal anti-inflammatory drugs during febrile attacks, showing only limited benefit [30]. Based on the pathophysiology of MKD, statins were thought to be useful although they resulted ineffective in the majority of cases [24, 30]. Many patients get better with corticosteroids, especially when given in high doses at the beginning of an attack [24]. However, when recurrent inflammatory attacks are very close, chronic high dose corticosteroid administration might lead to multiple side effects, such as early-onset osteoporosis. In these cases, anti-TNF-*α* and anti-IL-1 agents are reasonable therapeutic alternatives, though with variable results, confirming the complex and still unraveled cytokine dysregulation in MKD [21]. Noteworthy, the nitrogen-containing bisphosphonate alendronate has been reported to bring about an unexpected remission of all symptoms and laboratory abnormalities in a MKD patient [31].

*Tumor necrosis factor receptor-associated periodic syndrome* (TRAPS) is the most protean entity among HPFs in terms of age of disease onset, frequency, length, and severity of inflammatory attacks. Clinical features of TRAPS include prolonged recurrent fever episodes with periorbital edema, migratory erythematous plaques with underlying myalgia, arthralgia, and serous membranes inflammation; pericardial involvement has been also reported as the only manifestation of TRAPS, mimicking autoimmune disorders [32–43]. Although recurrent pericarditis is the most frequent cardiovascular manifestation, TRAPS mutations seem also to be responsible for early-onset atherosclerosis, thrombosis, and acute myocardial infarction [44, 45]. TRAPS heterogeneity is probably linked to the wide spectrum of* TNFRSF1A* mutations [46], affecting the interaction between TNF and its two transmembrane receptors (TNFR), characterized by four cysteine-rich extracellular domains, in a wide range of cell types. In physiological circumstances, TNFR extracellular domains can be cleaved, generating soluble receptor fragments that may function as natural TNF-antagonists. The majority of* TNFRSFA1* mutations affect the extracellular domain of TNFR, leading to changes in the three-dimensional structure of the ectodomains or alterations in ligand-independent TNFR assembly, with accumulation of the mutated receptors in the endoplasmic reticulum, final activation of proinflammatory signals, and prolonged survival of the activated inflammatory cells. Altered receptor cleavage (the “shedding hypothesis”) was evaluated initially to be a key-process in TRAPS, but this has not been demonstrated in many causing-disease* TNFRSF1A* mutations. In particular, R92Q is considered to be a low-penetrance mutation leading to later disease presentation, shorter-lasting febrile episodes, less frequent occurrence of rash and abdominal pain, and even potential spontaneous resolution. Genome-wide association studies have also identified the R92Q mutation as a genetic risk factor for multiple sclerosis and a susceptibility locus for vascular thrombosis in Behçet's disease, atherosclerosis, and other inflammatory phenomena [47, 48].

The average age of TRAPS onset is around 3 years; however, adult onset of TRAPS, up to the sixtieth decade, has been described as well and related to low-penetrance mutations. Patients with adult-onset TRAPS may be characterized by a phenotype that also mimics FMF in the duration of inflammatory attacks, that is, less than 1 week [32]. Diagnosis of TRAPS relies both on a compatible clinical phenotype and identification of a* TNFRSF1A* mutation [29]. Results of treatment are highly variable in TRAPS; a few patients gain some symptomatic relief from high-dose nonsteroidal anti-inflammatory drugs, while colchicine and different immunomodulators such as cyclosporine and thalidomide produce poor benefit [49]. Inflammatory attacks are often responsive to corticosteroid administration, but patients may become prone to metasteroidal comorbidities [50]. Furthermore, corticosteroids do not seem to provide complete protection from the risk of developing reactive amyloidosis [51]. Etanercept has been proven to induce a good disease control, although in some cases it gradually loses efficacy [45, 52–54]. Noteworthy, infliximab and adalimumab may paradoxically induce a typical acute inflammatory attack in TRAPS patients for reasons only partially understood [54, 55]. On the contrary, anti-IL-1 agents are reasonable treatment options in order to prevent disease relapses both in the short- and long-term [21, 22, 56].

**Figure 1:**
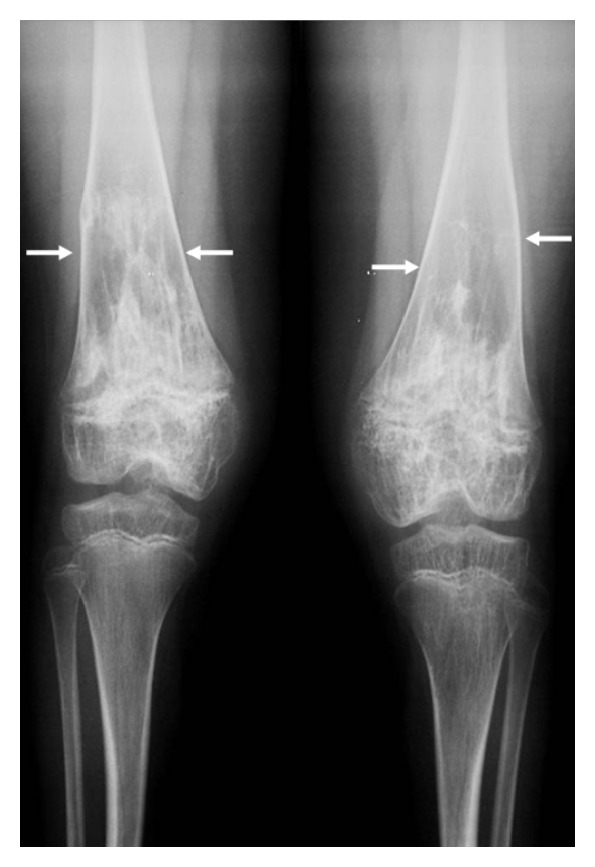
Anteroposterior radiograph performed in a 12-year-old boy with chronic infantile neurological cutaneous and articular (CINCA) syndrome, showing a calcified mass-like region in the physis of the distal femur (see white arrows). Osteoarthropathy of CINCA patients is a unique feature of this condition, caused by abnormal endochondral bone growth, mainly affecting large joints and long bones, beginning in the first infancy and causing striking changes until skeletal maturity.

*Cryopyrin-associated periodic syndrome* (CAPS) includes a spectrum of apparently distinct inflammatory disorders of increasing severity: FCAS, MWS, and CINCAs, all caused by* NLRP3* mutations, though the molecular basis for this distinction is still not well-understood [4, 29, 36]. FCAS is characterized by fever, urticaria-like rash, fatigue, arthralgia, myalgia, headache, and conjunctivitis, which usually appear after exposure to cold [57]. In addition to the clinical features seen in FCAS, MWS is also characterized by sensorineural hearing loss. As regards CINCAs, also called “neonatal onset multisystem inflammatory disease,” this is the most severe CAPS phenotype, characterized by diffuse subcontinuous neutrophilic urticaria-like erythema without pruritus occurring within a few days after birth, distinctive arthropathy with knee and patella deformity, chronic aseptic meningitis combined with ocular involvement (in terms of conjunctivitis, uveitis, retinopathy, and optic nerve atrophy, potentially causing blindness), bilateral sensorineural hearing loss, and progressive mental retardation [58].  Figure 1 shows typical CINCAs osteoarthropathy in a 12-years old boy. The extent of organ damage and permanent disability varies considerably among patients with FCAS, MWS, and CINCAs. Although CAPS are regularly marked by onset of symptoms during early infancy or childhood [59, 60], a case series of patients presenting with adult onset FCAS-like symptoms and carrying the low-penetrance Q703K mutation in the* NLRP3* gene has recently been described [61]. Consistent with CAPS pathogenesis, linked to the constitutively increased inflammasome activity and chronic oversecretion of IL-1*β*, lifelong treatment with IL-1 blocking therapies is the most logical option to control organ disease and damage; different strategies to block IL-1 have been used in clinical trials, all characterized by dramatic success in bringing down episodes of fever, urticaria-like rash, joint pain, and elevations in acute-phase reactants. Stabilization of the central nervous system inflammation with IL-1 antagonists has been sustained after several years of treatment even in CINCA patients [62, 63], and IL-1 blocking therapies have also permitted to prevent long-term development of systemic amyloidosis [21, 22]. The severity gradient of clinical signs from FCAS to CINCAs may be a consequence of the amount of IL-1 production, which could also explain the substantially higher doses of IL-1 blocking therapy needed to control inflammation in patients with CINCAs. About 30 to 50% of patients who present CAPS clinical picture, responding impressively to IL-1 blocking agents, are negative for any mutations in the* NLRP3* gene. Recent reports have shown the presence of somatic mosaicism in circulating leukocytes and epithelial cells of such “mutation-negative” patients; the extent to which mosaicism in mutation-negative cases can account for CAPS phenotype is a lively field of investigation [64].

Key-points and discriminating factors suggesting the diagnosis of specific HPFs included in systemic autoinflammatory diseases (SAIDs) are listed as follows:

 self-limited febrile attacks with localized symptoms (abdominal or thoracic pain, joint pain, rash, migratory muscular involvement, and periorbital edema);
 recurrent serositis;
 family history of similar attacks in horizontal or vertical distribution;
 peculiar ethnic origin;
 response to colchicine or corticosteroids;
 positive genetic test.

**Figure 2:**
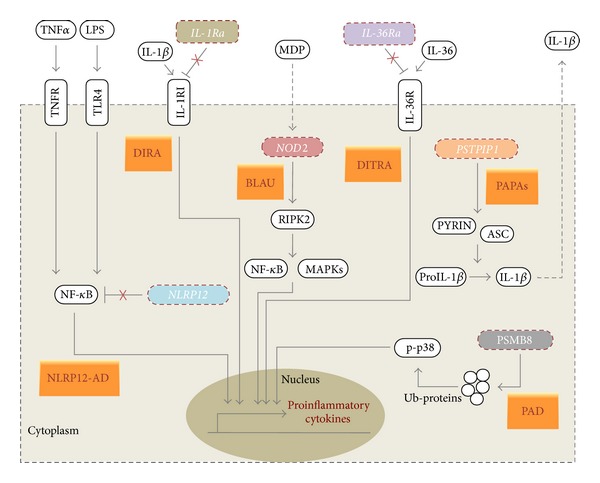
Schematic representation of the main pathophysiologic mechanisms involved in the less frequently diagnosed systemic autoinflammatory diseases. Blau syndrome (BLAU) and* NLRP12*-associated autoinflammatory disorder (NLRP12-AD) are caused by altered nuclear factor-*κ*B (NF-*κ*B) regulation, caused, respectively, by* NOD2* and* NLRP12* gene mutations. Deficiency of the interleukin-1 (IL-1) receptor antagonist (DIRA) and deficiency of interleukin-36 (IL-36) receptor antagonist (DITRA) are linked to mutations of genes coding, respectively, for IL-1 receptor antagonist (*IL-1Ra*) and IL-36 receptor antagonist (*IL-36Ra*). These mutations lead to the loss of IL-1*β* and IL-36 natural inhibition, resulting in uncontrolled proinflammatory responses. PAPA syndrome (PAPAs) is associated with increased IL-1*β* processing and secretion, as a consequence of proline-serine-threonine phosphatase-interacting protein 1 (*PSTPIP1*) gene mutations. Proteasome-associated diseases (PAD) are caused by mutations in the proteasome subunit beta type 8 (PSMB8), which lead to the accumulation of ubiquitinated proteins (Ub-proteins) and in turn to p38 phosphorylation, resulting in enhanced proinflammatory responses.

Although HPFs are the best-known clinical entities among SAIDs, in recent years numerous other less-known and still rarely identified disorders have been included in this emerging chapter of medicine.  Figure 2 shows a schematic representation of the main pathophysiologic mechanisms involved in the less frequently diagnosed SAIDs, revealing that mutated proteins cooperate in the regulation of the inflammasome activity, activating a proinflammatory cascade with IL-1*β* and TNF-*α* as prominent cytokines.

## 3. *NLRP12*-Associated Autoinflammatory Disorder

**Figure 3:**
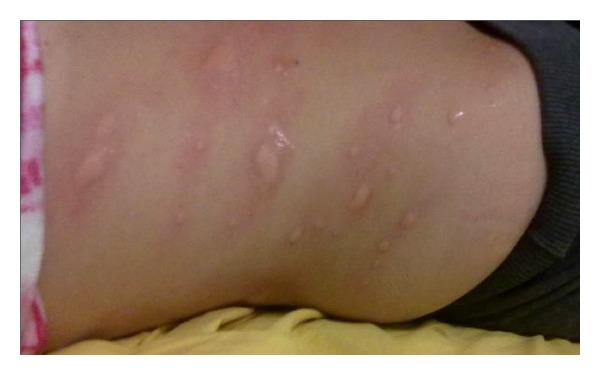
Cold-induced skin rash in a young patient with* NLRP12*-associated autoinflammatory disorder.

*NLRP12*-associated autoinflammatory disorder (NLRP12-AD) is a rare autosomal-dominant condition, firstly observed in two families originating from Guadeloupe, who presented a nonsense mutation in the* NLRP12 *gene, situated at the locus 19q13.4, encoding the monarch-1 protein, which is mainly expressed in monocytes and is involved in the activation of NF-*κ*B protein complex via canonical and noncanonical pathways [65, 66]. Disease onset usually occurs in the first year of life with recurrent high fevers and other inflammatory symptoms, mostly evoked by exposure to cold: urticaria-like skin rash, polyarthritis or arthralgia, myalgia, and headache.  Figure 3 represents an example of cold induced urticaria-like rash in a patient affected with NLRP12-AD. Currently, NLRP12-AD treatment is empirical and based on the severity of clinical manifestations. Corticosteroids and antihistamines during wintertime may bring about a good clinical response in the less severe cases; in addition, satisfactory therapeutic effects have been obtained with nonsteroidal anti-inflammatory drugs [67, 68]. It should be also noted that in a recent study two NLRP12-AD patients underwent anakinra treatment with only initial clinical improvement [69]. Based on this evidence, it was deduced that other proinflammatory cytokines, such as TNF-*α* and IL-6, could have a pathogenic role, suggesting the use of anti-TNF-*α* and anti-IL-6 drugs as potential alternative treatments [22].

## 4. Blau Syndrome

Blau syndrome (BS), first reported in 1985 as “juvenile systemic granulomatosis,” is a rare autosomal-dominant disease caused by* NOD2/CARD15* gene mutations; the gene was individuated in 2001 on the locus 16q12.1-13, which also contains the genetic susceptibility region for Crohn's disease and early-onset sarcoidosis (EOS) [70, 71], and codifies for NOD2, a sensor of bacterial antigens which induces NF-*κ*B activation [72]. Originally, BS was considered a separate entity from EOS, but it has been demonstrated that many individuals with EOS also present* NOD2/CARD15* mutations. For this reason, researchers have proposed distinguishing the familiar form, definitely named BS, from sporadic forms, which are called EOS [73, 74]. The disease can be observed in Caucasians and also in Asians and Afro-Americans [75]; in most patients the onset is around the 3rd-4th year of age with joint and skin granulomatous inflammatory signs; eye involvement usually develops later, between ages 7 and 12 [76–81]. Symmetrical polyarthritis with effusion in the little joints is the most common manifestation, frequently leading to finger deformities and ankylosis [82, 83]. More than 90% of patients have variable skin rashes, although ichthyosis-like changes are the most commonly observed [77]. Eye involvement, generally in the form of recurrent panuveitis, and often evolving towards chorioretinitis, glaucoma, and cataract, is the most significant danger in BS [84–86]. Moreover, the disease can also be revealed by granulomatous inflammatory processes affecting kidneys, liver, heart, and brain [87–90]. The presence of nonnecrotizing granulomas on synovial or skin biopsy should require the individuation of* NOD2/CARD15* mutations. Treatment of BS remains controversial; the prolonged administration of corticosteroids and immunosuppressant agents (methotrexate and cyclosporine) has led to variable results. Eye involvement usually responds adequately to low-dose corticosteroids during nonacute periods, while higher doses are required in acute attacks [84]. Biological agents such as infliximab or anakinra may be additional therapeutic choices, although in vitro studies seem to demonstrate that BS is not associated with IL-1 oversecretion [80, 91]. It is also interesting to note that a pilot study conducted on two patients with the sporadic form of EOS/BS showed that thalidomide mitigated granulomatous inflammation through a specific interference with differentiation of monocytes and inhibition of NF-*κ*B [92].

## 5. Pigmentary Hypertrichosis and Nonautoimmune Insulin-Dependent Diabetes Mellitus Syndrome

This syndrome (named also PHID syndrome) is an autosomal-recessive autoinflammatory disorder due to mutations in the* SLC29A3* gene, which codifies the human equilibrative nucleoside transporter-3 (hENT3), regulating apoptosis and macrophage recruitment [93]. Several studies have demonstrated that PHID inflammatory response is related to NF-*κ*B activation and cytokine overproduction. The clinical onset is in early childhood with pigmented skin lesions, hypertrichosis, and insulin-dependent diabetes mellitus without any autoantibodies. Other symptoms may include short stature, delayed puberty, and exocrine pancreatic insufficiency [94, 95]. Recently, PHID syndrome has also been associated with lipodystrophy, abnormal distribution of perivisceral fat, scleroderma-like skin modifications, and cardiomyopathy resulting from chronic inflammation, resistant to anti-TNF-*α* and anti-IL-1 therapy [94]. In regard to treatment, adalimumab and anakinra have produced little benefit in controlling systemic inflammation of PHID syndrome, while new therapeutic horizons might derive from the use of agents acting directly against macrophages, such as etoposide [95].

## 6. Proteasome-Associated Disorders

The ubiquitin-proteasome system participates in the qualitative control and degradation of different proteins, also playing an active role in cell cycle regulation, DNA repair, and signal transduction mechanisms [96, 97]. A dysregulated proteolytic activity in the proteasome is associated with numerous syndromes, sharing different features of SAIDs [98]. In particular, mutations in the* PSMB8* gene, located at the locus 6p21 and codifying the inducible subunit *β* type-8 (PSMB8) of the proteasome, have been identified as the cause of Nakajo-Nishimura syndrome [99–101], recently redefined as “Japanese autoinflammatory syndrome with lipodystrophy” [102] and known in Western countries as “joint contractures, muscle atrophy, and panniculitis-induced lipodystrophy syndrome” (or JMP) [103, 104]. This group of disorders also includes CANDLE, standing for “chronic atypical neutrophilic dermatitis with lipodystrophy and elevated temperature,” syndrome [105–107].

*Nakajo-Nishimura syndrome* (NNS) is a very rare disorder starting in infancy with pernio-like rashes during the first cold months, gradually evolving into lipodystrophy mainly in the face and upper extremities and remittent fevers, firstly described in 1939 in Japan [99, 100, 108]; a distinct homozygous* PSMB8* mutation encoding the proteasome *β*5i subunit has been identified as its genetic cause, leading to reduced proteolytic activity and accumulation of ubiquitin proteins. The presence of long clubbed fingers and hypertrophic periostosis are other common NNS features. As regards treatment, skin lesions regress with high-dose corticosteroids but tend to reappear when they are tapered. The use of corticosteroids has no effect, however, on lipodystrophy, and even worsens central obesity, while biological agents, such as anti-TNF-*α* and anti-IL-1*β*, might be working alternatives [109]. Although NNS has been assumed to be found exclusively in Japan, a similar syndrome related to* PSMB8* mutations has been reported from foreign countries and named JMP [103, 110].

*CANDLE syndrome* has been also described as distinct from NNS, though determined by missense mutations of the same* PSMB8* gene and other proteasome-associated genes [105]; it starts during the first year of life with recurrent fevers as high as 42°C, lymphadenopathy, arthralgia, purplish eyelid edema, abnormal growth of lips, and progressive lipodystrophy [111]. Interferon-*γ* is the key-molecule in CANDLE syndrome, and a clinical trial is in progress to test the use of baricitinib, Janus kinase JAK1 and JAK2 inhibitor, to deregulate interferon production and allow corticosteroid dose reduction [112].

## 7. Pyogenic Autoinflammatory Diseases

SAIDs presenting with nonbacterial pyogenic manifestations include pyogenic arthritis, pyoderma gangrenosum and acne (PAPA) syndrome, deficiency of the IL-1-receptor antagonist, deficiency of the IL-36 receptor antagonist, Majeed syndrome, and chronic recurrent multifocal osteomyelitis.

*PAPA syndrome* (PAPAs) is an autosomal-dominant condition caused by mutations in the* PSTPIP1* gene, situated at the locus 15q24-q25.1, which codifies the CD2-binding protein 1 (CD2BP1), enclosed in the pyrin-mediated inflammatory pathway [113]. In the case of a* PSTPIP1* mutation, the CD2BP1-pyrin link is facilitated; hence, the percentage of CD2BP1 binding to pyrin is higher, determining a proinflammatory state [114]. PAPAs starts in the first or second decade and is characterized by sterile pyogenic pauciarticular arthritis, cystic acne, ulcerative lesions occurring on the lower extremities, and pyogenic abscesses at the injection sites; cultures from the synovial liquid and skin lesions are characteristically negative [115]. Corticosteroid therapy brings about inconstant success, while anti-TNF-*α* (etanercept, infliximab) and anti-IL-1 (anakinra) agents usually give positive results in corticosteroid-resistant patients [116–118]. Recently, two patients have been described with manifestations similar to PAPA syndrome but without joint involvement; in both cases, there was also the presence of hidradenitis suppurativa in the armpits. Although these patients did not bear any* PSTPIP1* mutations, they presented CCTG microsatellites repetitions in the promoter region of the* PSTPIP1* gene; this condition, characterized by the triad of pyoderma gangrenosum, acne, and hidradenitis suppurativa, has been distinguished from the “classic” PAPAs and identified with the acronym PASH (pyoderma gangrenosum, acne, and suppurativa hidradenitis) syndrome [119].

*Deficiency of the IL-1-receptor antagonist* (DIRA) is an autosomal-recessive disorder secondary to homozygous missense and nonsense loss-of-function mutations in the* IL1RN* gene, located on the long arm of chromosome 2, which codifies the IL-1 receptor antagonist [120], physiologically antagonizing the effect of the proinflammatory cytokines IL-1*α* and IL-1*β*. Aksentijevich et al. identified* IL1RN* mutations in 9 individuals, all displaying an abnormal function of the IL-1 receptor antagonist protein and leading to uninhibited signaling of IL-1 [121]. This condition starts at birth and is characterized by multifocal osteomyelitis, periostitis with heterotopic nuclei of ossification, pustular, and/or ichthyosis skin lesions. Patients with DIRA respond only partially to high-dose corticosteroids, while anti-IL-1 treatment with anakinra appears the most obvious and effective therapy, which gives rapid and prolonged clinical remission [122].

A recently reported disease has been named with the acronym DITRA, standing for* deficiency of the IL-36 receptor antagonist*, as it depends on mutations in the* IL36R* gene, located on chromosome 2, which encodes the IL-36 receptor antagonist, predominantly expressed in keratinocytes but not in fibroblasts and endothelial cells, having the role of inhibiting the activation of NF-*κ*B [123]. Clinical manifestations, occasionally triggered by viral or bacterial infections, as pustulous skin lesions, high fever (until 40–42°C), arthralgia, glossitis with “geographic tongue,” and asthenia, arise during infancy [124]. To date, there are no consolidated therapies for the cure of this pathology; however, acitretin, an oral retinoid usually used for psoriasis and hidradenitis suppurativa, has been effective in most patients, while corticosteroids, methotrexate, cyclosporine, or anakinra can give diverse results in terms of efficacy [125].

*Majeed syndrome* is a rare autosomal recessive clinical entity, first identified in 1989, caused by mutations in the* LPIN2* gene, localized on the short arm of chromosome 18, which codifies the lipin-2 protein, expressed in liver, kidney, gastrointestinal tract, lymphatic tissue, and bone marrow [126]. Lipin-2 regulates proinflammatory signals determined by saturated fatty acids, and its mutated variant leads to chronic recurrent multifocal osteomyelitis, congenital dyserythropoietic anemia, and neutrophilic dermatosis, usually around the second year of life [127, 128]. Skin lesions start with erythematosus papules and well-defined painful plaques all over the body, often resembling Sweet's syndrome or psoriasis. Considering the rarity of the syndrome, treatment is still empirical, nonsteroidal anti-inflammatory drugs may be useful, while corticosteroids can be used for short periods to control both osteomyelitis and skin signs. Recently, some authors have described a significant radiological improvement after administration of anakinra and canakinumab in Majeed syndrome, while the lack of response to anti-TNF-*α* agents (etanercept) in these same patients suggests the presence of yet-to-be-clarified pathogenetic mechanisms [129].

*Chronic recurrent multifocal osteomyelitis* encompasses a group of disorders under the acronym “CRMO” described for the first time by Giedion et al. in 1972 [130], and manifesting with autoinflammatory attacks predominantly affecting the metaphysis and epiphysis of long bones, each attack is characterized by severe bone pain, increased osteoclastic activity, and bone remodeling [131]. The average onset age is around 10 years, and females are more frequently affected than males. The sites most often involved are shinbones, followed by pelvis, proximal femora, clavicles, and heels [132]. Permanent physical invalidity, bone deformities, or limb dysmetria might result from untreated or overlooked CRMO. Different bone pathologies require a strict differential diagnosis, such as infections (septic osteomyelitis, typical, and atypical mycobacterial infections), neoplasms (malignant primitive bone tumors, osteoid osteoma, leukemias/lymphomas), metabolic disorders, osteopetrosis, and other SAIDs (as PAPAs). The fact that individuals from the same family may be affected by CRMO suggests a genetic susceptibility to the disease combined with heterogeneous polygenic influences [133, 134]. There is no gold-standard treatment for CRMO, which has long been based on nonsteroidal anti-inflammatory drugs, like indomethacin and glucocorticoids, which must be limited to the short-term, especially in adolescents, due to the risk of side effects impacting growth [135, 136]. Actually, the most frequently adopted treatment is centred on bisphosphonates. Their efficacy was first reported in 2004 [137] and confirmed by several clinical studies published later [138]. TNF-*α* antagonists have also been employed with good results in the few CRMO patients refractory to bisphosphonates and nonsteroidal anti-inflammatory drugs [139].

## 8. Conclusive Remarks

The increasingly accessible genetic investigation techniques currently available have led to and probably will continue to deliver the identification of a greater number of rare genetic diseases belonging to the web of SAIDs. The discovery of new disorders involving the innate immunity system and specifically related to inflammasome dysfunction, accompanied by intensive molecular and clinical research activity, will increase our knowledge in the basic mechanisms of inflammation. In the end, genotype-phenotype correlations will help us in the most proper evaluation of genetic tests on one hand and in the individuation of diagnostic pathways and adequate therapies on the other.

## Figures and Tables

**Table 1:** Brief summary of clinical features of systemic autoinflammatory diseases.

Disease	Gene locus	Protein	Inheritance	Clinical features	Treatment
FMF	*MEFV* 16p13.3	Pyrin (marenostrin)	AR	Fever, serositis, arthralgias or arthritides, erysipelas-like eruption on the legs, and systemic amyloidosis in untreated or noncompliant patients	Colchicine, anakinra, and canakinumab
MKD	*MVK* 12q24	Mevalonate kinase	AR	Fever, polymorphous rash, arthralgias, abdominal pain, diarrhea, lymph node enlargement, splenomegaly, and aphthosis	NSAIDs, corticosteroids, anakinra, and anti-TNF-*α* agents
TRAPS	*TNFRSF1A* 12p13	Tumor necrosis factor receptor 1	AD	Fever, migratory muscle and joint involvement, conjunctivitis, periorbital edema, arthralgias or arthritis, serosal involvement, corticosteroid responsiveness of inflammatory attacks, and risk of amyloidosis	Corticosteroids, etanercept, and anakinra
FCAS	*NLRP3* 1q44	Cryopyrin	AD	Fever, cold-induced urticaria-like rash, conjunctivitis, and arthralgias	Anakinra, rilonacept, and canakinumab
MWS				Fever, urticaria-like rash, conjunctivitis and episcleritis, arthralgias, neurosensorial deafness, and amyloidosis	
CINCAs				Subcontinuous fever, early-onset urticaria-like rash, clubbing, corneal clouding, anterior or posterior uveitis, papilledema, retinopathy with scarring, optic nerve atrophy, aseptic chronic meningitis, increased intracranial pressure, inner ear inflammation with neurosensorial deafness, deforming osteoarthritis involving large joints, bony overgrowth, joint contractures, severe growth retardation with facial dysmorphic features (frontal bossing and flattening of the nasal bridge), and amyloidosis	
NLRP12-AD	*NLRP12* 19q13	Monarch-1	AD	Fever, arthralgia, cold-induced urticaria-like rash, abdominal complaint, and risk of sensorineural deafness	Antihistamines, NSAIDs, anakinra, anti-TNF-*α* agents, and IL-6 receptor antagonists
BLAUs	*NOD2* (*CARD15*) 16q12.1-13	NOD2	AD	Granulomatous dermatitis with ichthyosis-like changes, symmetrical granulomatous polyarthritis, recurrent granulomatous panuveitis, risk of cranial neuropathies, and intermittent fever	Corticosteroids, immunosuppressive agents, anti-TNF-*α* agents, and thalidomide
PHIDs	*SLC29A3* 10q22	hENT3	AR	Fever, pigmented skin lesions, hypertrichosis, insulin-dependent diabetes mellitus, pancreatic insufficiency, cardiomyopathy, lipodystrophy, scleroderma-like lesions, short stature, and delayed puberty	Etoposide?
NNS	*PSMB8* 6p21	Inducible subunit *β* of the proteasome	AR	Fever, long clubbed fingers and toes with joint contractures, lipomuscular atrophy, pernio-like rash in hands and feet, heliotrope rash on the eyelids, nodular skin lesions, basal ganglia calcification, and hepatosplenomegaly	Corticosteroids, anti-TNF-*α* agents, and anti-IL-1 agents
CANDLEs				Recurrent fever, arthralgia, purplish skin lesions, abnormal growth of lips, lipodystrophy, hypertrichosis, acanthosis nigricans, alopecia areata, nodular episcleritis, conjunctivitis, chondritis of the nose and ear, aseptic meningitis, and basal ganglia calcification	Corticosteroids, anti-TNF-*α* agents, IL-6 receptor antagonists, and baricitinib
PAPAs	*PSTPIP1* 15q24-q25.1	CD2BP1	AD	Pauciarticular pyogenic arthritis, osteocartilaginous erosions of joints, cystic acne, ulcerative lesions of lower limb extremities, and pyogenic abscesses	Corticosteroids, etancercept, infliximab, and anakinra
DIRA	*IL1RN* 2q14.2	IL-1 receptor antagonist	AR	Neonatal multifocal osteomyelitis, periostitis with osteolytic lesions, pustulous, and ichthyosis skin rash	Anakinra and corticosteroids
DITRA	*IL36R* 2q14	IL-36 receptor antagonist	AR	Fever, pustulous skin lesions on the palms and soles, glossitis, arthritis, severe bone pain, and asthenia	Corticosteroids, methotrexate, cyclosporine, anakinra, and acitretin
MAJEEDs	*LPIN2* 18p11.31	Lipin-2	AR	Recurrent multifocal osteomyelitis, congenital dyserythropoietic anemia, neutrophilic dermatosis with palmoplantar pustulosis, or pyoderma gangrenosum	NSAIDs, corticosteroids, anakinra, and canakinumab
CRMO	Unknown, presumably polygenetic trait		Currently unknown	Osteomyelitis, bone pain with localized osteolysis, potential association with Sweet's syndrome, acne, or inflammatory bowel disease, recurrent fever	NSAIDs, corticosteroids, bisphosphonates (pamidronate, neridronate), and anti-TNF-*α* agents (infliximab)

AD: autosomal dominant; AR: autosomal recessive; BLAUs: Blau syndrome; CANDLEs: chronic atypical neutrophilic dermatosis with lipodystrophy and elevated temperature; CINCAs: chronic infantile neurologic cutaneous articular syndrome; CRMO: chronic recurrent multifocal osteomyelitis; DIRA: deficiency of the interleukin-1-receptor antagonist; DITRA: deficiency of the IL-36 receptor antagonist; FCAS: familial cold autoinflammatory syndrome; FMF: familial Mediterranean fever; MAJEEDs: Majeed syndrome; MKD: mevalonate kinase deficiency syndrome; MWS: Muckle-Wells syndrome; NLRP12-AD: *NLRP12*-associated autoinflammatory disorder; NNS: Nakajo-Nishimura syndrome; NSAIDs: nonsteroidal anti-inflammatory drugs; PAPAs: pyogenic arthritis, pyoderma gangrenosum, and acne syndrome; PHIDs: pigmentary hypertrichosis and nonautoimmune insulin-dependent diabetes mellitus syndrome; TRAPS: tumor necrosis factor receptor-associated periodic syndrome.

**Table 2:** Main clues useful for differential diagnosis of hereditary periodic fever syndromes.

	Familial Mediterranean fever	Mevalonate kinase deficiency syndrome	Tumor necrosis factor receptor-associated periodic syndrome	Cryopyrin-associated periodic syndrome
Onset	Childhood or adolescence	Infancy (first year of life)	3–20 years	Neonatal period-childhood
Usual ethnicity	Armenian, nonsephardic Jews, Arab, Turkish people	Dutch and other Northern European populations	Firstly recognized in Northern European (Ireland and Scotland) people; any ethnicity	Panethnic
Fever duration	1–4 days	3–7 days	1 or even 3-4 weeks, usually responding to corticosteroids	Subcontinuous/variable with circadian periodism and intermittent flares
Abdominal distress	Very common (in the form of sterile peritonitis)	Very common (abdominal pain, vomiting, diarrhea)	Common (abdominal pain, diarrhea, constipation)	Uncommon
Chest involvement	Pleurisy, often unilateral	Infrequent	Pleuritis	Absent
Skin involvement	Erysipelas-like rash on feet/ankles	Polymorphic rash, erythema elevatum diutinum, disseminated superficial actinic porokeratosis	Painful migratory eruption, edematous plaques	Neutrophilic urticaria-like skin eruption of variable severity and extension (either induced by cold exposure or constant)
Osteoarticular involvement	Arthralgias or arthritides	Arthralgias	Migratory arthralgias, nonerosive arthritides	Arthralgias of variable severity, deforming osteoarthritis involving large joints and contiguous bones (in CINCA syndrome)

## References

[1] Lamkanfi M, Walle LV, Kanneganti T-D (2011). Deregulated inflammasome signaling in disease. *Immunological Reviews*.

[2] Drenth JPH, Van Der Meer JWM (2006). The inflammasome—a linebacker of innate defense. *New England Journal of Medicine*.

[3] Obici L, Merlini G (2012). Amyloidosis in autoinflammatory syndromes. *Autoimmunity Reviews*.

[4] Masters SL, Simon A, Aksentijevich I, Kastner DL (2009). Horror autoinflammaticus: the molecular pathophysiology of autoinflammatory disease. *Annual Review of Immunology*.

[5] Lidar M, Livneh A (2007). Familial mediterranean fever: clinical, molecular and management advancements. *Netherlands Journal of Medicine*.

[6] Majeed HA, Shahin HM, Ghandour K (2000). The acute scrotum in Arab children with familial Mediterranean fever. *Pediatric Surgery International*.

[7] Ozen S, Demirkaya E, Amaryan G (2014). Results from a multicentre international registry of familial Mediterranean fever: impact of environment on the expression of a monogenic disease in children. *Annals of the Rheumatic Diseases*.

[8] Senel K, Melikoglu MA, Baykal T, Melikoglu M, Erdal A, Ugur M (2010). Protracted febrile myalgia syndrome in familial Mediterranean fever. *Modern Rheumatology*.

[9] Sohar E, Gafni J, Pras M, Heller H (1967). Familial Mediterranean fever. A survey of 470 cases and review of the literature. *The American Journal of Medicine*.

[10] Cantarini L, Capecchi PL, Lucherini OM, Laghi Pasini F, Galeazzi M (2010). Familial Mediterranean fever diagnosed in an elderly patient. *Clinical and Experimental Rheumatology*.

[11] Sayarlioglu M, Cefle A, Inanc M (2005). Characteristics of patients with adult-onset familial Mediterranean fever in Turkey: analysis of 401 cases. *International Journal of Clinical Practice*.

[12] Livneh A, Langevitz P, Zemer D (1997). Criteria for the diagnosis of familial Mediterranean fever. *Arthritis and Rheumatism*.

[13] Ece A, Cakmak E, Uluca U (2014). The MEFV mutations and their clinical correlations in children with familial Mediterranean fever in southeast Turkey. *Rheumatology International*.

[14] Grateau G, Pêcheux C, Cazeneuve C (2000). Clinical versus genetic diagnosis of familial Mediterranean fever. *Oxford Journals Medicine*.

[15] Cazeneuve C, Hovannesyan Z, Geneviève D (2003). Familial Mediterranean fever among patients from Karabakh and the diagnostic value of MEFV gene analysis in all classically affected populations. *Arthritis and Rheumatism*.

[16] Booty MG, Jae JC, Masters SL (2009). Familial Mediterranean fever with a single MEFV mutation: where is the second hit?. *Arthritis and Rheumatism*.

[17] Booth DR, Gillmore JD, Lachmann HJ (2000). The genetic basis of autosomal dominant familial Mediterranean fever. *Oxford Journals Medicine*.

[18] Rigante D, La Torraca I, Avallone L, Pugliese AL, Gaspari S, Stabile A (2006). The pharmacologic basis of treatment with colchicine in children with familial Mediterranean fever. *European Review for Medical and Pharmacological Sciences*.

[19] Ben-Chetrit E, Levy M (1998). Familial Mediterranean fever. *The Lancet*.

[20] Ben-Chetrit E, Bergmann S, Sood R (2006). Mechanism of the anti-inflammatory effect of colchicine in rheumatic diseases: a possible new outlook through microarray analysis. *Rheumatology*.

[21] Haar NT, Lachmann H, Özen S (2013). Treatment of autoinflammatory diseases: results from the Eurofever Registry and a literature review. *Annals of the Rheumatic Diseases*.

[22] Vitale A, Rigante D, Lucherini OM (2013). Biological treatments: new weapons in the management of monogenic autoinflammatory disorders. *Mediators of Inflammation*.

[23] Steichen O, Van Der Hilst J, Simon A, Cuisset L, Grateau G (2009). A clinical criterion to exclude the hyperimmunoglobulin D syndrome (mild mevalonate kinase deficiency) in patients with recurrent fever. *Journal of Rheumatology*.

[24] Van Der Hilst JCH, Bodar EJ, Barron KS (2008). Long-term follow-up, clinical features, and quality of life in a series of 103 patients with hyperimmunoglobulinemia D syndrome. *Medicine*.

[25] Rigante D, Capoluongo E, Bertoni B (2007). First report of macrophage activation syndrome in hyperimmunoglobulinemia D with periodic fever syndrome. *Arthritis and Rheumatism*.

[26] Siemiatkowska AM, van den Born LI, van Hagen PM (2013). Mutations in the mevalonate kinase (*MVK*) gene cause nonsyndromic retinitis pigmentosa. *Ophthalmology*.

[27] Zhang S-Q, Jiang T, Li M (2012). Exome sequencing identifies *MVK* mutations in disseminated superficial actinic porokeratosis. *Nature Genetics*.

[28] Cantarini L, Rigante D, Brizi MG (2012). Clinical and biochemical landmarks in systemic autoinflammatory diseases. *Annals of Medicine*.

[29] Rigante D (2012). The fresco of autoinflammatory diseases from the pediatric perspective. *Autoimmunity Reviews*.

[30] Bader-Meunier B, Florkin B, Sibilia J (2011). Mevalonate kinase deficiency: a survey of 50 patients. *Pediatrics*.

[31] Cantarini L, Vitale A, Magnotti F, Lucherini OM, Caso F, Frediani B (2013). Weekly oral alendronate in mevalonate kinase deficiency. *Orphanet Journal of Rare Diseases*.

[32] Dodé C, André M, Bienvenu T (2002). The enlarging clinical, genetic, and population spectrum of tumor necrosis factor receptor-associated periodic syndrome. *Arthritis and Rheumatism*.

[33] Rigante D, Frediani B, Galeazzi M, Cantarini L (2013). From the mediterranean to the sea of Japan: the transcontinental odyssey of autoinflammatory diseases. *BioMed Research International*.

[34] Trost S, Rosé CD (2005). Myocarditis and sacroiliitis: 2 Previously unrecognized manifestations of tumor necrosis factor receptor associated periodic syndrome. *Journal of Rheumatology*.

[35] Schmaltz R, Vogt T, Reichrath J (2010). Skin manifestations in tumor necrosis factor receptor-associated periodic syndrome (TRAPS). *Dermato-Endocrinology*.

[36] Tchernitchko D, Chiminqgi M, Galactéros F (2005). Unexpected high frequency of P46L *TNFRSF1A* allele in sub-Sahara West African populations. *European Journal of Human Genetics*.

[37] Cantarini L, Lucherini OM, Cimaz R, Galeazzi M (2010). Recurrent pericarditis caused by a rare mutation in the *TNFRSF1A* gene and with excellent response to anakinra treatment. *Clinical and Experimental Rheumatology*.

[38] Cantarini L, Lucherini OM, Brucato A (2012). Clues to detect tumor necrosis factor receptor-associated periodic syndrome (TRAPS) among patients with idiopathic recurrent acute pericarditis: results of a multicentre study. *Clinical Research in Cardiology*.

[39] Cantarini L, Lucherini OM, Baldari CT, Laghi Pasini F, Galeazzi M (2010). Familial clustering of recurrent pericarditis may disclose tumour necrosis factor receptor-associated periodic syndrome. *Clinical and Experimental Rheumatology*.

[40] Cantarini L, Lucherini OM, Cimaz R (2009). Idiopathic recurrent pericarditis refractory to colchicine treatment can reveal tumor necrosis factor receptor-associated periodic syndrome. *International Journal of Immunopathology and Pharmacology*.

[41] Cantarini L, Lucherini OM, Iacoponi F (2010). Development and preliminary validation of a diagnostic score for identifying patients affected with adult-onset autoinflammatory disorders. *International Journal of Immunopathology and Pharmacology*.

[42] Cantarini L, Iacoponi F, Lucherini OM (2011). Validation of a diagnostic score for the diagnosis of autoinflammatory diseases in adults. *International Journal of Immunopathology and Pharmacology*.

[43] Magnotti F, Vitale A, Rigante D (2013). The most recent advances in pathophysiology and management of tumour necrosis factor receptor associated periodic syndrome (TRAPS): personal experience and literature review. *Clinical and Experimental Rheumatology*.

[44] Chia S, Qadan M, Newton R, Ludlam CA, Fox KAA, Newby DE (2003). Intra-arterial tumor necrosis factor-*α* impairs endothelium-dependent vasodilatation and stimulates local tissue plasminogen activator release in humans. *Arteriosclerosis, Thrombosis, and Vascular Biology*.

[45] Stojanov S, Dejaco C, Lohse P (2008). Clinical and functional characterisation of a novel *TNFRSF1A* c.605T>A/V173D cleavage site mutation associated with tumour necrosis factor receptor-associated periodic fever syndrome (TRAPS), cardiovascular complications and excellent response to etanercept treatment. *Annals of the Rheumatic Diseases*.

[46] Kimberley FC, Lobito AA, Siegel RM, Screaton GR (2007). Falling into TRAPS—receptor misfolding in the TNF receptor 1-associated periodic fever syndrome. *Arthritis Research and Therapy*.

[47] Caminero A, Comabella M, Montalban X (2011). Role of tumour necrosis factor (TNF)-*α* and *TNFRSF1A* R92Q mutation in the pathogenesis of TNF receptor-associated periodic syndrome and multiple sclerosis. *Clinical and Experimental Immunology*.

[48] Amoura Z, Dodé C, Hue S (2005). Association of the R92Q *TNFRSF1A* mutation and extracranial deep vein thrombosis in patients with Behçet’s disease. *Arthritis and Rheumatism*.

[49] Hull KM, Drewe E, Aksentijevich I (2002). The TNF receptor-associated periodic syndrome (TRAPS): emerging concepts of an autoinflammatory disorder. *Medicine*.

[50] McDermott MF, Aksentijevich I, Galon J (1999). Germline mutations in the extracellular domains of the 55 kDa TNF receptor, TNFR1, define a family of dominantly inherited autoinflammatory syndromes. *Cell*.

[51] Aganna E, Hawkins PN, Ozen S (2004). Allelic variants in genes associated with hereditary periodic fever syndromes as susceptibility factors for reactive systemic AA amyloidosis. *Genes and Immunity*.

[52] Cantarini L, Rigante D, Lucherini OM (2010). Role of etanercept in the treatment of tumor necrosis factor receptor-associated periodic syndrome: personal experience and review of the literature. *International Journal of Immunopathology and Pharmacology*.

[53] Gattorno M, Pelagatti MA, Meini A (2008). Persistent efficacy of anakinra in patients with tumor necrosis factor receptor-associated periodic syndrome. *Arthritis and Rheumatism*.

[54] Drewe E, Powell RJ, Mcdermott EM (2007). Comment on: failure of anti-TNF therapy in TNF Receptor 1-Associated Periodic Syndrome (TRAPS). *Rheumatology*.

[55] Nedjai B, Hitman GA, Quillinan N (2009). Proinflammatory action of the antiinflammatory drug infliximab in tumor necrosis factor receptor-associated periodic syndrome. *Arthritis and Rheumatism*.

[56] Brizi MG, Galeazzi M, Lucherini OM, Cantarini L, Cimaz R (2012). Successful treatment of tumor necrosis factor receptor-associated periodic syndrome with canakinumab. *Annals of Internal Medicine*.

[57] Yu JR, Leslie KS (2011). Cryopyrin-associated periodic syndrome: an update on diagnosis and treatment response. *Current Allergy and Asthma Reports*.

[58] Cantarini L, Lucherini OM, Frediani B (2011). Bridging the gap between the clinician and the patient with cryopyrin-associated periodic syndromes. *International Journal of Immunopathology and Pharmacology*.

[59] Goldbach-Mansky R, Dailey NJ, Canna SW (2006). Neonatal-onset multisystem inflammatory disease responsive to interleukin-1*β* inhibition. *New England Journal of Medicine*.

[60] Neven B, Callebaut I, Prieur A-M (2004). Molecular basis of the spectral expression of *CIAS1* mutations associated with phagocytic cell-mediated autoinflammatory disorders CINCA/NOMID, MWS, and FCU. *Blood*.

[61] Vitale A, Lucherini OM, Galeazzi M, Frediani B, Cantarini L (2012). Long-term clinical course of patients carrying the Q703K mutation in the *NLRP3* gene: a case series. *Clinical and Experimental Rheumatology*.

[62] Agostini L, Martinon F, Burns K, McDermott MF, Hawkins PN, Tschopp J (2004). NALP3 forms an IL-1*β*-processing inflammasome with increased activity in Muckle-Wells autoinflammatory disorder. *Immunity*.

[63] Rigante D, Ansuini V, Caldarelli M, Bertoni B, La Torraca I, Stabile A (2006). Hydrocephalus in CINCA syndrome treated with anakinra. *Child’s Nervous System*.

[64] Goldbach-Mansky R (2011). Current status of understanding the pathogenesis and management of patients with NOMID/CINCA. *Current Rheumatology Reports*.

[65] Williams KL, Lich JD, Duncan JA (2005). The CATERPILLER protein Monarch-1 is an antagonist of toll-like receptor-, tumor necrosis factor *α*-, and Mycobacterium tuberculosis-induced pro-inflammatory signals. *Journal of Biological Chemistry*.

[66] Bonizzi G, Karin M (2004). The two NF-*κ*B activation pathways and their role in innate and adaptive immunity. *Trends in Immunology*.

[67] Borghini S, Tassi S, Chiesa S (2011). Clinical presentation and pathogenesis of cold-induced autoinflammatory disease in a family with recurrence of an *NLRP12* mutation. *Arthritis and Rheumatism*.

[68] Jéru I, Duquesnoy P, Fernandes-Alnemri T (2008). Mutations in NALP12 cause hereditary periodic fever syndromes. *Proceedings of the National Academy of Sciences of the United States of America*.

[69] Jéru I, Hentgen V, Normand S (2011). Role of interleukin-1*β* in *NLRP12*-associated autoinflammatory disorders and resistance to anti-interleukin-1 therapy. *Arthritis and Rheumatism*.

[70] Blau EB (1985). Familial granulomatous arthritis, iritis, and rash. *Journal of Pediatrics*.

[71] Cavanaugh J (2006). *NOD2*: ethnic and geographic differences. *World Journal of Gastroenterology*.

[72] Miceli-Richard C, Lesage S, Rybojad M (2001). *CARD15* mutations in Blau syndrome. *Nature Genetics*.

[73] Kanazawa N, Okafuji I, Kambe N (2005). Early-onset sarcoidosis and *CARD15* mutations with constitutive nuclear factor-*κ*B activation: common genetic etiology with Blau syndrome. *Blood*.

[74] Rosé CD, Doyle TM, McIlvain-Simpson G (2005). Blau syndrome mutation of *CARD15*/*NOD2* in sporadic early onset granulomatous arthritis. *Journal of Rheumatology*.

[75] Rybicki BA, Maliarik MJ, Bock CH (1999). The Blau syndrome gene is not a major risk factor for sarcoidosis. *Sarcoidosis Vasculitis and Diffuse Lung Disease*.

[76] Wang X, Kuivaniemi H, Bonavita G (2002). *CARD15* mutations in familial granulomatosis syndromes: a study of the original Blau syndrome kindred and other families with large-vessel arteritis and cranial neuropathy. *Arthritis and Rheumatism*.

[77] Becker ML, Rose CD (2005). Blau syndrome and related genetic disorders causing childhood arthritis. *Current rheumatology reports*.

[78] Milman N, Ursin K, Rødevand E, Nielsen FC, Hansen TVO (2009). A novel mutation in the *NOD2* gene associated with Blau syndrome a Norwegian family with four affected members. *Scandinavian Journal of Rheumatology*.

[79] Rosé CD, Wouters CH, Meiorin S (2006). Pediatric granulomatous arthritis: an international registry. *Arthritis and Rheumatism*.

[80] Aróstegui JI, Arnal C, Merino R (2007). *NOD2* gene-associated pediatric granulomatous arthritis: clinical diversity, novel and recurrent mutations, and evidence of clinical improvement with interleukin-1 blockade in a Spanish cohort. *Arthritis and Rheumatism*.

[81] Okafuji I, Nishikomori R, Kanazawa N (2009). Role of the *NOD2* genotype in the clinical phenotype of Blau syndrome and early-onset sarcoidosis. *Arthritis and Rheumatism*.

[82] Manouvrier-Hanu S, Puech B, Piette F (1998). Blau syndrome of granulomatous arthritis, iritis, and skin rash: a new family and review of the literature. *The American Journal of Medical Genetics*.

[83] Raphael SA, Blau EB, Hsu SH (1993). Analysis of a large kindred with Blau syndrome for HLA, autoimmunity, and sarcoidosis. *The American Journal of Diseases of Children*.

[84] Latkany PA, Jabs DA, Smith JR (2002). Multifocal choroiditis in patients with familial juvenile systemic granulomatosis. *The American Journal of Ophthalmology*.

[85] Kurokawa T, Kikuchi T, Ohta K, Imai H, Yoshimura N (2003). Ocular manifestations in Blau syndrome associated with a *CARD15*/*NOD2* mutation. *Ophthalmology*.

[86] Snyers B, Dahan K (2006). Blau syndrome associated with a *CARD15*/*NOD2* mutation. *The American Journal of Ophthalmology*.

[87] Saini SK, Rose CD (1996). Liver involvement in familial granulomatous arthritis (Blau syndrome). *Journal of Rheumatology*.

[88] See Ting S, Ziegler J, Fischer E (1998). Familial granulomatous arthritis (Blau syndrome) with granulomatous renal lesions. *Journal of Pediatrics*.

[89] Scerri L, Cook LJ, Jenkins EA, Thomas AL (1996). Familial juvenile systemic granulomatosis (Blau’s syndrome). *Clinical and Experimental Dermatology*.

[90] Mourad F, Tang A (2010). Sinus of valsalva aneurysm in Blau’s syndrome. *Journal of Cardiothoracic Surgery*.

[91] Milman N, Andersen CB, Hansen A (2006). Favourable effect of TNF-*α* inhibitor (infliximab) on Blau syndrome in monozygotic twins with a de novo *CARD15* mutation. *APMIS*.

[92] Yasui K, Yashiro M, Tsuge M (2010). Thalidomide dramatically improves the symptoms of early-onset sarcoidosis/blau syndrome: its possible action and mechanism. *Arthritis and Rheumatism*.

[93] Cliffe ST, Kramer JM, Hussain K (2009). *SLC29A3* gene is mutated in pigmented hypertrichosis with insulin-dependent diabetes mellitus syndrome and interacts with the insulin signaling pathway. *Human Molecular Genetics*.

[94] Melki I, Lambot K, Jonard L (2013). Mutation in the *SLC29A3* gene: a new cause of a monogenic, autoinflammatory condition. *Pediatrics*.

[95] Senniappan S, Hughes M, Shah P, Shah V, Kaski JP, Brogan P (2013). Pigmentary hypertrichosis and non-autoimmune insulin-dependent diabetes mellitus (PHID) syndrome is associated with severe chronic inflammation and cardiomyopathy, and represents a new monogenic autoinflammatory syndrome. *Journal of Pediatric Endocrinology and Metabolism*.

[96] Tanaka K (2009). The proteasome: overview of structure and functions. *Proceedings of the Japan Academy B*.

[97] Fehling HJ, Swat W, Laplace C (1994). MHC class I expression in mice lacking the proteasome subunit LMP-7. *Science*.

[98] Schmidt M, Finley D (2014). Regulation of proteasome activity in health and disease. *Biochimica et Biophysica Acta*.

[99] Kitano Y, Matsunaga E, Morimoto T, Okada N, Sano S (1985). A syndrome with nodular erythema, elongated and thickened fingers, and emaciation. *Archives of Dermatology*.

[100] Tanaka M, Miyatani N, Yamada S (1993). Hereditary lipo-muscular atrophy with joint contracture, skin eruptions and hyper-gamma-globulinemia: a new syndrome. *Internal Medicine*.

[101] Kasagi S, Kawano S, Nakazawa T (2008). A case of periodic-fever-syndrome-like disorder with lipodystrophy, myositis, and autoimmune abnormalities. *Modern Rheumatology*.

[102] Kitamura A, Maekawa Y, Uehara H (2011). A mutation in the immunoproteasome subunit PSMB8 causes autoinflammation and lipodystrophy in humans. *Journal of Clinical Investigation*.

[103] Garg A, Hernandez MD, Sousa AB (2010). An autosomal recessive syndrome of joint contractures, muscular atrophy, microcytic anemia, and panniculitis-associated lipodystrophy. *Journal of Clinical Endocrinology and Metabolism*.

[104] Mégarbané A, Sanders A, Chouery E, Delague V, Medlej-Hashim M, Torbey P-H (2002). An unknown autoinflammatory syndrome associated with short stature and dysmorphic features in a young boy. *Journal of Rheumatology*.

[105] Torrelo A, Patel S, Colmenero I (2010). Chronic atypical neutrophilic dermatosis with lipodystrophy and elevated temperature (CANDLE) syndrome. *Journal of the American Academy of Dermatology*.

[106] Ramot Y, Czarnowicki T, Maly A, Navon-Elkan P, Zlotogorski A (2011). Chronic atypical neutrophilic dermatosis with lipodystrophy and elevated temperature syndrome: a case report. *Pediatric Dermatology*.

[107] Oyanagi K, Sasaki K, Ohama E (1987). An autopsy case of a syndrome with muscular atrophy, decreased subcutaneous fat, skin eruption and hyper *γ*-globulinemia: peculiar vascular changes and muscle fiber degeneration. *Acta Neuropathologica*.

[108] Kanazawa N (2012). Nakajo-Nishimura syndrome: an autoinflammatory disorder showing pernio-like rashes and progressive partial lipodystrophy. *Allergology International*.

[109] Arima K, Kinoshita A, Mishima H (2011). Proteasome assembly defect due to a proteasome subunit beta type 8 (PSMB8) mutation causes the autoinflammatory disorder, Nakajo-Nishimura syndrome. *Proceedings of the National Academy of Sciences of the United States of America*.

[110] Agarwal AK, Xing C, Demartino GN (2010). PSMB8 encoding the *β*5i proteasome subunit is mutated in joint contractures, muscle atrophy, microcytic anemia, and panniculitis-induced lipodystrophy syndrome. *The American Journal of Human Genetics*.

[111] Kluk J, Rustin M, Brogan PA (2014). CANDLE syndrome: a report of a novel mutation and review of the literature. *The British Journal of Dermatology*.

[112] Montealegre Sanchez GA, Reinhardt AL, Brogan P (2013). Chronic atypical neutrophilic dermatosis with lipodystrophy and elevated temperatures (CANDLE): clinical characterization and initial response to Janus kinase inhibition with baricitinib. *Arthritis & Rheumatology*.

[113] Shoham NG, Centola M, Mansfield E (2003). Pyrin binds the PSTPIP1/CD2BP1 protein, defining familial Mediterranean fever and PAPA syndrome as disorders in the same pathway. *Proceedings of the National Academy of Sciences of the United States of America*.

[114] Wise CA, Gillum JD, Seidman CE (2002). Mutations in CD2BP1 disrupt binding to PTP PEST and are responsible for PAPA syndrome, an autoinflammatory disorder. *Human Molecular Genetics*.

[115] Yeon HB, Lindor NM, Seidman JG, Seidman CE (2000). Pyogenic arthritis pyoderma gangrenosum, and acne syndrome maps to chromosome 15q. *The American Journal of Human Genetics*.

[116] Cortis E, De Benedetti F, Insalaco A (2004). Abnormal production of the tumor necrosis factor inhibitor etanercept and clinical efficacy of tumor in a patient with PAPA syndrome. *Journal of Pediatrics*.

[117] Stichweh DS, Punaro M, Pascual V (2005). Dramatic improvement of pyoderma gangrenosum with infliximab in a patient with PAPA syndrome. *Pediatric Dermatology*.

[118] Dierselhuis MP, Frenkel J, Wulffraat NM, Boelens JJ (2005). Anakinra for flares of pyogenic arthritis in PAPA syndrome. *Rheumatology*.

[119] André MFJ, Aumaître O, Grateau G (2010). Longest form of CCTG microsatellite repeat in the promoter of the CD2BP1/PSTPIP1 gene is associated with aseptic abscesses and with crohn disease in French patients. *Digestive Diseases and Sciences*.

[120] Reddy S, Jia S, Geoffrey R (2009). An autoinflammatory disease due to homozygous deletion of the IL1RN locus. *New England Journal of Medicine*.

[121] Aksentijevich I, Masters SL, Ferguson PJ (2009). An autoinflammatory disease with deficiency of the interleukin-1-receptor antagonist. *New England Journal of Medicine*.

[122] Schnellbacher C, Ciocca G, Menendez R (2013). Deficiency of interleukin-1 receptor antagonist responsive to anakinra. *Pediatric Dermatology*.

[123] Marrakchi S, Guigue P, Renshaw BR (2011). Interleukin-36-receptor antagonist deficiency and generalized pustular psoriasis. *New England Journal of Medicine*.

[124] Onoufriadis A, Simpson MA, Pink AE (2011). Mutations in *IL36RN/IL1F5* are associated with the severe episodic inflammatory skin disease known as generalized pustular psoriasis. *The American Journal of Human Genetics*.

[125] Rossi-Semerano L, Piram M, Chiaverini C, De Ricaud D, Smahi A, Koné-Paut I (2013). First clinical description of an infant with interleukin-36-receptor antagonist deficiency successfully treated with anakinra. *Pediatrics*.

[126] Majeed HA, Kalaawi M, Mohanty D (1989). Congenital dyserythropoietic anemia and chronic recurrent multifocal osteomyelitis in three related children and the association with Sweet syndrome in two siblings. *The Journal of Pediatrics*.

[127] Valdearcos M, Esquinas E, Meana C (2012). Lipin-2 reduces proinflammatory signaling induced by saturated fatty acids in macrophages. *Journal of Biological Chemistry*.

[128] Al-Mosawi ZS, Al-Saad KK, Ijadi-Maghsoodi R, El-Shanti HI, Ferguson PJ (2007). A splice site mutation confirms the role of LPIN2 in Majeed syndrome. *Arthritis and Rheumatism*.

[129] Herlin T, Fiirgaard B, Bjerre M (2013). Efficacy of anti-IL-1 treatment in Majeed syndrome. *Annals of the Rheumatic Diseases*.

[130] Giedion A, Holthusen W, Masel LF, Vischer D (1972). Subacute and chronic “symmetrical” osteomyelitis. *Annales de Radiologie*.

[131] Coinde E, David L, Cottalorda J (2001). Chronic recurrent multifocal osteomyelitis in children: a report of 17 cases. *Archives de Pediatrie*.

[132] Khanna G, Sato TSP, Ferguson P (2009). Imaging of chronic recurrent Multifocal Osteomyelitis. *Radiographics*.

[133] Hurtado-Nedelec M, Chollet-Martin S, Chapeton D, Hugot J-P, Hayem G, Gérard B (2010). Genetic susceptibility factors in a cohort of 38 patients with SAPHO syndrome: a study of *PSTPIP2, NOD2*, and *LPIN2* genes. *Journal of Rheumatology*.

[134] Beck C, Girschick HJ, Morbach H (2011). Mutation screening of the IL-1 receptor antagonist gene in chronic non-bacterial osteomyelitis of childhood and adolescence. *Clinical and Experimental Rheumatology*.

[135] Abril JC, Ramirez A (2007). Successful treatment of chronic recurrent multifocal osteomyelitis with indomethacin: a preliminary report of five cases. *Journal of Pediatric Orthopaedics*.

[136] Girschick HJ, Zimmer C, Klaus G, Darge K, Dick A, Morbach H (2007). Chronic recurrent multifocal osteomyelitis: what is it and how should it be treated?. *Nature Clinical Practice Rheumatology*.

[137] Kerrison C, Davidson JE, Cleary AG, Beresford MW (2004). Pamidronate in the treatment of childhood SAPHO syndrome. *Rheumatology*.

[138] Miettunen PMH, Wei X, Kaura D, Reslan WA, Aguirre AN, Kellner JD (2009). Dramatic pain relief and resolution of bone inflammation following pamidronate in 9 pediatric patients with persistent chronic recurrent multifocal osteomyelitis (CRMO). *Pediatric Rheumatology*.

[139] Marangoni RG, Halpern ASR (2010). Chronic recurrent multifocal osteomyelitis primarily affecting the spine treated with Anti-TNF therapy. *Spine*.

